# Author Correction: Cross-species comparison reveals therapeutic vulnerabilities halting glioblastoma progression

**DOI:** 10.1038/s41467-025-65072-9

**Published:** 2025-10-17

**Authors:** Leo Carl Foerster, Oguzhan Kaya, Valentin Wüst, Diana-Patricia Danciu, Vuslat Akcay, Milica Bekavac, Kevin Chris Ziegler, Nina Stinchcombe, Anna Tang, Susanne Kleber, Jocelyn L. Y. Tang, Jan Brunken, Irene Lois-Bermejo, Noelia Gesteira-Perez, Xiujian Ma, Ahmed Sadik, Phuong Uyen Le, Kevin Petrecca, Christiane A. Opitz, Haikun Liu, Christian Rainer Wirtz, Angela Goncalves, Anna Marciniak-Czochra, Simon Anders, Ana Martin-Villalba

**Affiliations:** 1 Molecular Neurobiology, German Cancer Research Center (DKFZ), Heidelberg, Germany; 2 Combined Faculty of Mathematics, Engineering and Natural Sciences, University of Heidelberg, Heidelberg, Germany; 3 BioQuant Centre, University of Heidelberg, Heidelberg, Germany; 4 Institute of Mathematics, University of Heidelberg, Heidelberg, Germany; 5 Molecular Neurogenetics, German Cancer Research Center (DKFZ), Heidelberg, Germany; 6 Metabolic Crosstalk in Cancer, German Cancer Research Center (DKFZ) and German Cancer Consortium (DKTK), DKFZ Core Center Heidelberg, Heidelberg, Germany; 7 Department of Neurosciences, Montreal Neurological Institute-Hospital, McGill University, Montreal, QC Canada; 8 Department of Neurosurgery, Ulm University Hospital, Ulm, Germany; 9 Computational and Molecular Prevention, German Cancer Research Center (DKFZ), Heidelberg, Germany; 10 DKFZ Hector Cancer Institute, University Medicine Mannheim, Heidelberg, Germany; 11 Medical Faculty Mannheim, University of Heidelberg, Heidelberg, Germany; 12 Interdisciplinary Center for Scientific Computing, University of Heidelberg, Heidelberg, Germany

**Keywords:** Neural stem cells, CNS cancer, Computational biology and bioinformatics

Correction to: *Nature Communications* 10.1038/s41467-025-62528-w, published online 06 August 2025

The original version of this Article contained an error in the spelling of the author Jocelyn L.Y. Tang, which was incorrectly given as Joceyln Tang. This has now been corrected in both the PDF and HTML versions of the Article.

