# Supplementary material for: Cross-species comparison reveals therapeutic vulnerabilities halting glioblastoma progression
Source: Nat Commun. 2025 Aug 6;16:7250. doi: 10.1038/s41467-025-62528-w (PMC12329047; doi:10.1038/s41467-025-62528-w)
Supplement: Supplementary file 1 — Supplementary Information [file 41467_2025_62528_MOESM1_ESM.pdf]

Supplementary Information

for

Cross-species Comparison Reveals Therapeutic Vulnerabilities Halting Glioblastoma Progression

Leo Carl Foerster*, Oguzhan Kaya*, Valentin Wüst, Diana-Patricia Danciu, Irene Lois-Bermejo, Vuslat Akcay, Milica Bekavac, Kevin Chris Ziegler, Nina Stinchcombe, Anna Tang, Jan Brunken, Noelia Gesteira Perez, Xiujian Ma, Ahmed Sadik, Christiane Opitz, Haikun Liu, Christian Rainer Wirtz, Anna Marciniak-Czochra, Simon Anders, Angela Goncalves, and Ana Martin-Villalba

Content:

8 Supplemental Figures

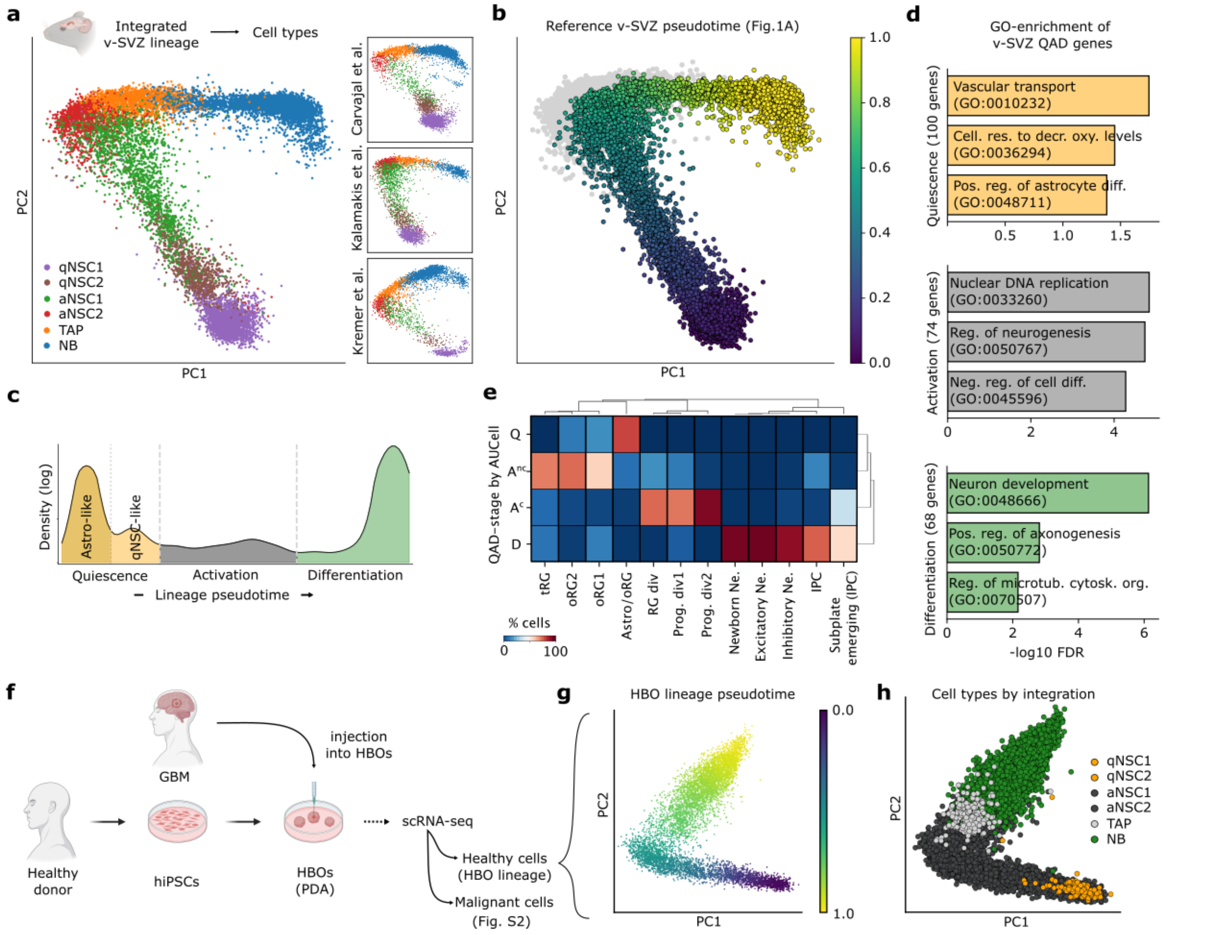

Supplementary Fig. 1 | Adult murine v-SVZ NSC lineage QAD stages and their counterpart in human cortical development

a Left: PCA of integrated v-SVZ NSC lineage cells from Fig. 1a with cell types from [1] obtained by label transfer. Right: lineage datasets[1–3] with consistent transitions captured by PCA. **b** Diffusion pseudotime fit on v-SVZ NSC lineage PCA in (a), used as the NSC reference pseudotime. Cycling cells are colored gray. **c** Pseudotime cell density for NSC lineage from (b) by Gaussian KDE, with valleys denoting boundaries between QAD-stages. The Q-stage is subset into dormant astrocyte-like (Astro-like) and qNSC-like subsets based on the separation of qNSC1 and qNSC2 in (a). **d** Selected GO-enrichments for SVZ-QAD genes by cell stage. P-values from hypergeometric test with FDR-correction. **e** Overlap of QAD-stage and cycling cells with selected cell types from the human cortical development meta-atlas[4]. QAD-stage was determined by AUCell score-max. **f** Scheme of the generation of PDA tumors by injection of GBM cells into HBOs. Healthy (see Supplementary Fig. 3) and malignant (see Supplementary Fig. 2) populations are identified by inferCNV (Methods). Created in BioRender. Kaya, O. (2025) https://BioRender.com/np19rtk. **g-h** PCA of the integrated HBO NSC lineage dataset with diffusion pseudotime (g) and cell types by integration (h). Note similarities to lineage stages in (a-b). NB: neuroblast; TAP: transit amplifying progenitor; RG: radial glia; Ne.: neuron; IPC: intermediate progenitor cell; PDA: patient-derived allograft; HBO: human brain organoid; hiPSC: human induced pluripotent stem cell. Source data are provided as a Source Data file.

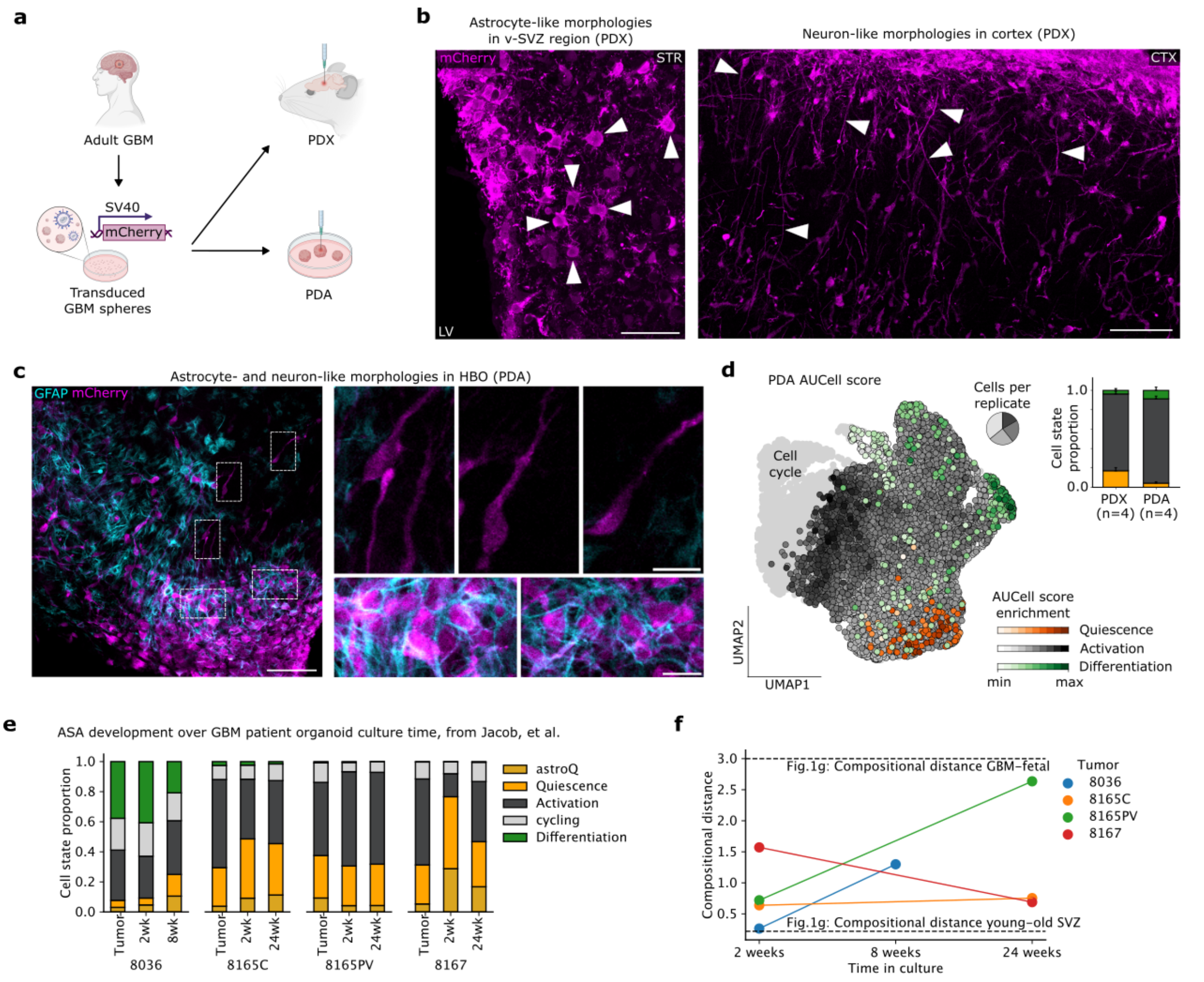

Supplementary Fig. 2 | QAD stage inference across modalities and time

a Generation of T6 PDX tumors by orthotopic injection into mouse brains. TCF/Lef-EGFP reports canonical Wnt signaling while mCherry ubiquitously labels tumor cells. Created in BioRender. Kaya, O. (2025) https://BioRender.com/np19rtk. **b** Representative immunofluorescence images of GBM cells in a T6 PDX, 5.5 mpi. Arrowheads highlight tumor cell morphologies in the v-SVZ (left) and cortex (right). Scale bars 100µm. **c** Representative immunofluorescence images of GBM cells in a T6 PDA, 2 wpi, with tumor cell morphologies highlighted. mCherry labels GBM cells. Scale bars 100µm, in insets 25µm. **d** AUCell scoring of T6 PDA cells (n=4 replicates) as in (c). Cycling cells are colored gray. Inset barchart depicts related ASA between PDX (Fig. 1f) and PDA contexts. Bars present mean, error bars standard deviation among n=4 replicates. **e** Stacked barcharts depicting the proportion of QAD-stage cells in primary tumor tissue (Tumor) as well as 2, 8, and 24 weeks (wk) in culture. Data are from Jacob et al[5]. **f** Compositional distances between tumors from (e) and corresponding *in vitro* culture times indicate early establishment of ASA which remains temporally stable. For reference, horizontal dotted lines denote compositional differences from Fig. 1g, as indicated. PDX: patient derived xenograft; PDA: patient-derived allograft; mpi: months post-injection; wpi: weeks post-injection; STR: striatum; LV: lateral ventricle; CTX: cortex; HBO: human brain organoid. Source data are provided as a Source Data file.

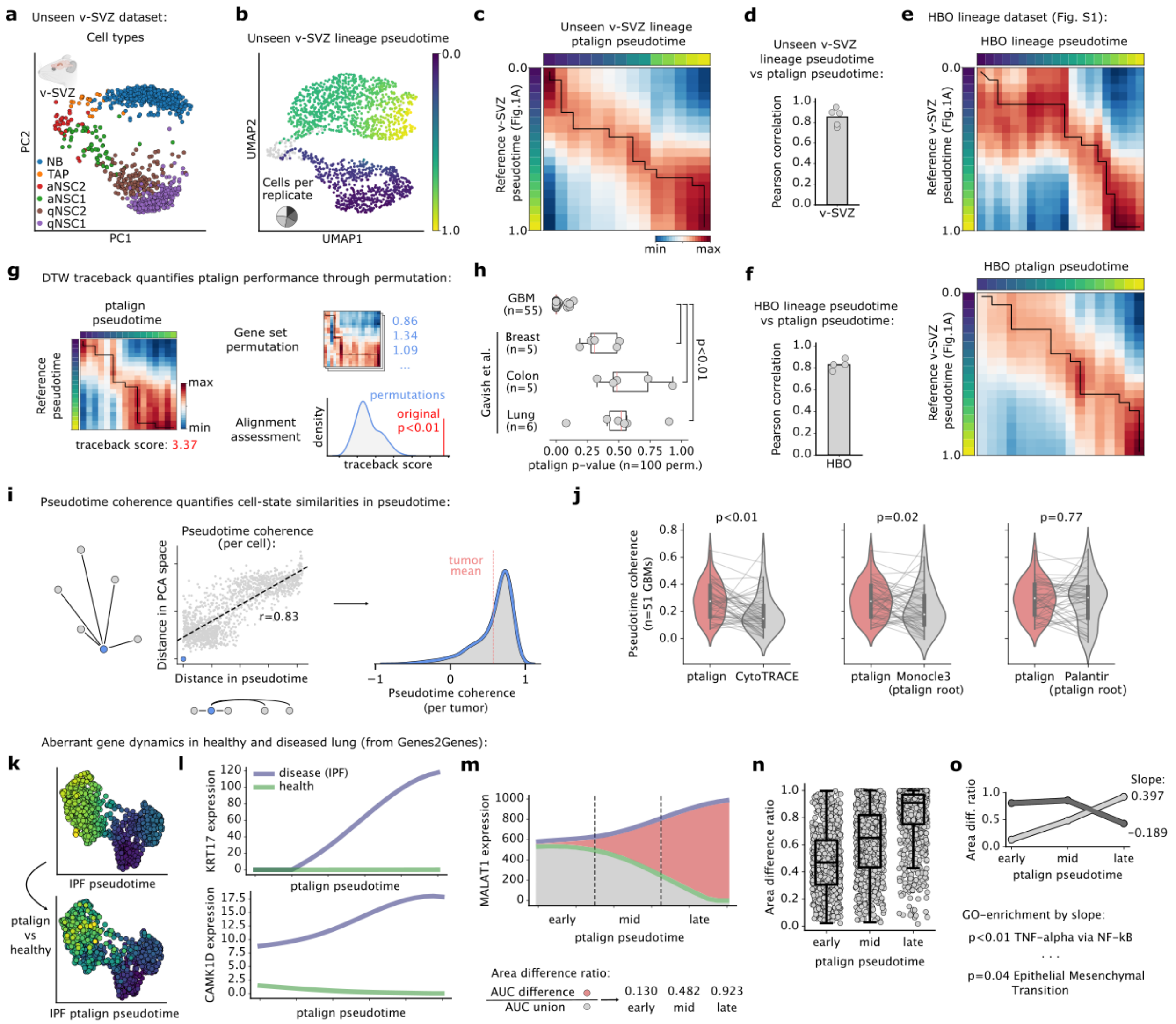

a Unseen v-SVZ dataset: Cell types
v-SVZ
NB
TAP
aNSC2
aNSC1
qNSC2
qNSC1
PC1
PC2
b Unseen v-SVZ lineage pseudotime
Cells per replicate
UMAP1
UMAP2
c Unseen v-SVZ lineage ptalign pseudotime
Reference v-SVZ pseudotime (Fig.1A)
min
max
d Unseen v-SVZ lineage pseudotime vs ptalign pseudotime:
Pearson correlation
v-SVZ
e HBO lineage dataset (Fig. S1):
HBO lineage pseudotime
Reference v-SVZ pseudotime (Fig.1A)
f HBO lineage pseudotime vs ptalign pseudotime:
Pearson correlation
HBO
HBO ptalign pseudotime
g DTW traceback quantifies ptalign performance through permutation:
ptalign pseudotime
Reference pseudotime
traceback score: 3.37
Gene set permutation
0.86
1.34
1.09
Alignment assessment
permutations
original
p<0.01
density
traceback score
h
GBM (n=55)
Gavish et al.
Breast (n=5)
Colon (n=5)
Lung (n=6)
p<0.01
ptalign p-value (n=100 perm.)
i Pseudotime coherence quantifies cell-state similarities in pseudotime:
Pseudotime coherence (per cell):
Distance in PCA space
Distance in pseudotime
r=0.83
tumor mean
Pseudotime coherence (per tumor)
j
Pseudotime coherence (n=51 GBMs)
p<0.01
p=0.02
p=0.77
ptalign
CytoTRACE
Monocle3 (ptalign root)
Palantir (ptalign root)
Aberrant gene dynamics in healthy and diseased lung (from Genes2Genes):
k
IPF pseudotime
ptalign vs healthy
IPF ptalign pseudotime
l
disease (IPF)
health
KRT17 expression
CAMK1D expression
ptalign pseudotime
m
MALAT1 expression
early
mid
late
ptalign pseudotime
Area difference ratio:
AUC difference
AUC union
0.130 early
0.482 mid
0.923 late
n
Area difference ratio
early
mid
late
ptalign pseudotime
o
Area diff. ratio
Slope:
0.397
−0.189
early
mid
late
ptalign pseudotime
GO-enrichment by slope:
p<0.01 TNF-alpha via NF-kB
p=0.04 Epithelial Mesenchymal Transition

Supplementary Fig. 3 | ptalign benchmarking by dataset and algorithm

a Expression PCA from n=5 replicates of an unseen v-SVZ NSC lineage dataset with cell types by label transfer. **b** UMAP of the unseen v-SVZ NSC lineage from (a) with diffusion pseudotime. Cycling cells are colored gray. **c** DTW denoting lineage similarity between the unseen v-SVZ NSC lineage pseudotime from (b) and the reference v-SVZ NSC lineage from Fig. 1a. DTW values represent pseudotime-binned cell's Pearson correlation. **d** Pearson correlation in unseen v-SVZ lineage pseudotime (b) and ptalign pseudotime per replicate. **e** Top: DTW of HBO NSC lineage diffusion pseudotime (Supplementary Fig. 1) and the reference v-SVZ NSC lineage pseudotime (Fig. 1a). Bottom: DTW comparing the HBO ptalign pseudotime and v-SVZ NSC reference. **f** Analogous to (d) for HBO lineage diffusion pseudotime and ptalign pseudotime. **g** Permutation approach used to quantify ptalign performance. A DTW-score maximizing matrix traceback is compared for the original QAD and expression-matched permuted gene sets (Methods) to derive a permutation p-value. **h** ptalign permutation p-values for 55 primary GBM scRNA-seq datasets (Fig. 1) and other tissue tumors[6]. **i** The pseudotime coherence metric compares distances in PCA space and pseudotime for each tumor. The correlation between each tumor cell (blue) and all other tumor cells (gray) is computed per cell, then averaged to derive a coherence score per tumor (right). **j** Pseudotime coherence values for ptalign pseudotimes and named algorithms on 55 GBMs from (h). Scores for individual GBMs are linked. Statistical significance was assessed by paired t-test. **k** Top: Pseudotime for IPF cells and, Bottom: ptalign pseudotime for IPF cells aligned to healthy lung reference from [7]. Bottom: ptalign pseudotime for IPF cells aligned to healthy lung reference. **l** Expression splines for selected genes with variable dynamics in healthy and IPF lung, as reported in Genes2Genes[7] on the ptalign pseudotime. **m-n** Area difference ratio in pseudotime increments exemplified by MALAT1 (m) and shown across genes (n). **o** Slope of area difference ratio over pseudotime increments represents fluctuating expression divergence. GSEA by slope reveals enriched EMT genes among positive slopes. P-values from GSEA enrichment are FDR-adjusted. HBO: human brain organoid; DTW: dynamic time warping; IPF: idiopathic pulmonary fibrosis. Box plots in h, j, n span 25th to 75th percentile, with median indicated. Whiskers extend to 1.5 times the interquartile-range. Source data are provided as a Source Data file.

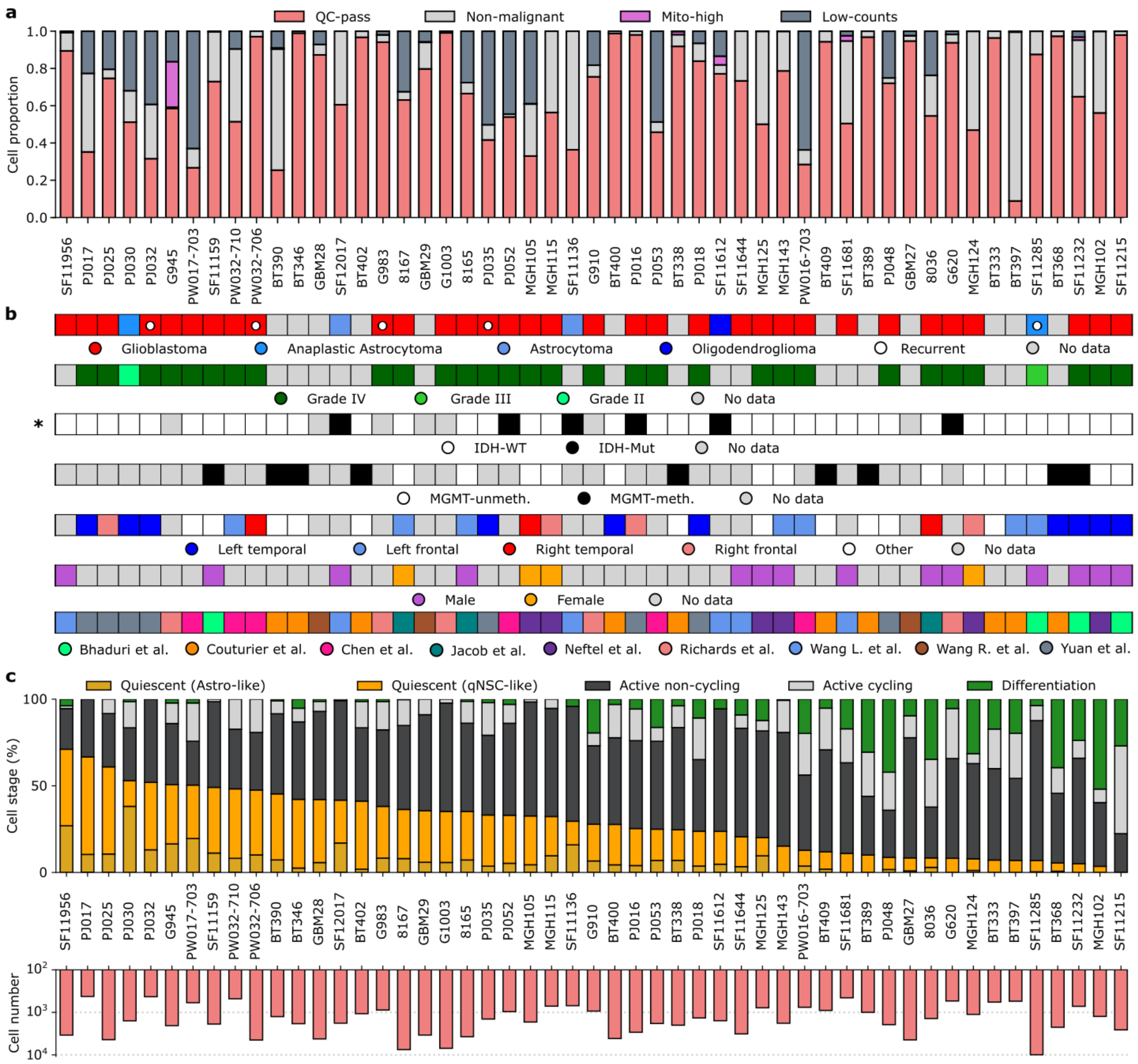

Supplementary Fig. 4 | External GBM scRNA-seq datasets metadata and ASA

a Barcharts relating proportion of cells filtered in QC-steps for published primary GBM scRNA-seq datasets (n=51, Supplementary Data 2) that pass ptalign permutation threshold. **b** Collated patient metadata across studies for tumor diagnosis, stage, IDH-mutation status, MGMT-methylation status, location, and sex. Asterisk (*) indicates significant association with QAD-stage by ANOVA. **c** Top: stacked barcharts depicting the proportion of cells assigned to QAD stages from ptalign pseudotimes, with Q-stage cells subset by Astro-like and qNSC-like stages. Cycling cells are colored gray. Bottom: number of cells assigned a ptalign pseudotime for each tumor. Each column in A-C relates information from the same tumor. Source data are provided as a Source Data file.

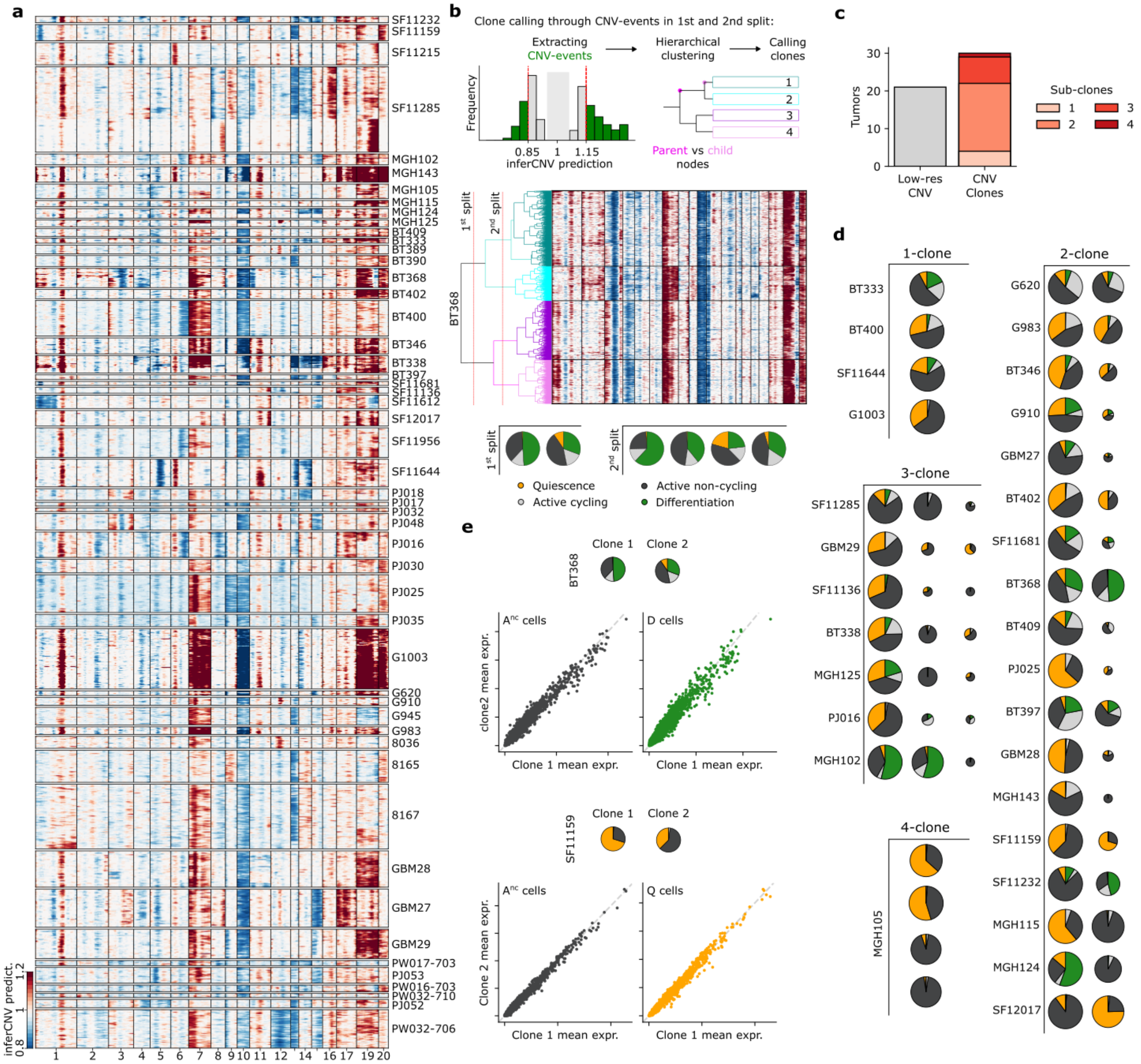

Supplementary Fig. 5 | CNV clones exhibit consistent QAD-stage transcriptomics

a Copy number variation (CNV) landscape by inferCNV of n=51 primary GBM tumors. CNV losses (blue) and gains (red) are arranged by chromosome (columns) and separated by tumor into cells (rows). Cells per tumor were hierarchically clustered by CNV-profile, with each 5th cell shown (of n=109,079 total). Sex chromosomes are not shown. **b** Schematic representation of clone assignment for tumor BT368. CNV-events are determined by thresholding inferCNV predictions and used to compare parent vs. child nodes in the 1st and 2nd split of a CNV-based hierarchical clustering to derive 1-4 clones per tumor (see Methods). **c** Barchart depicting CNV-clones assigned for individual GBMs. Clone assignment in Low-res tumors was inconclusive. **d** Proportion of QAD-stage and cycling cells per clone per tumor. Pie chart size relates relative clone size. **e** Gene expression in QAD-stage cells from two clones in BT368 (top) and SF11159 (bottom) denoted by pie charts. Scatterplots depict mean log-normalized expression of pseudobulk QAD-stage cells per clone, for all expressed genes. Source data are provided as a Source Data file.

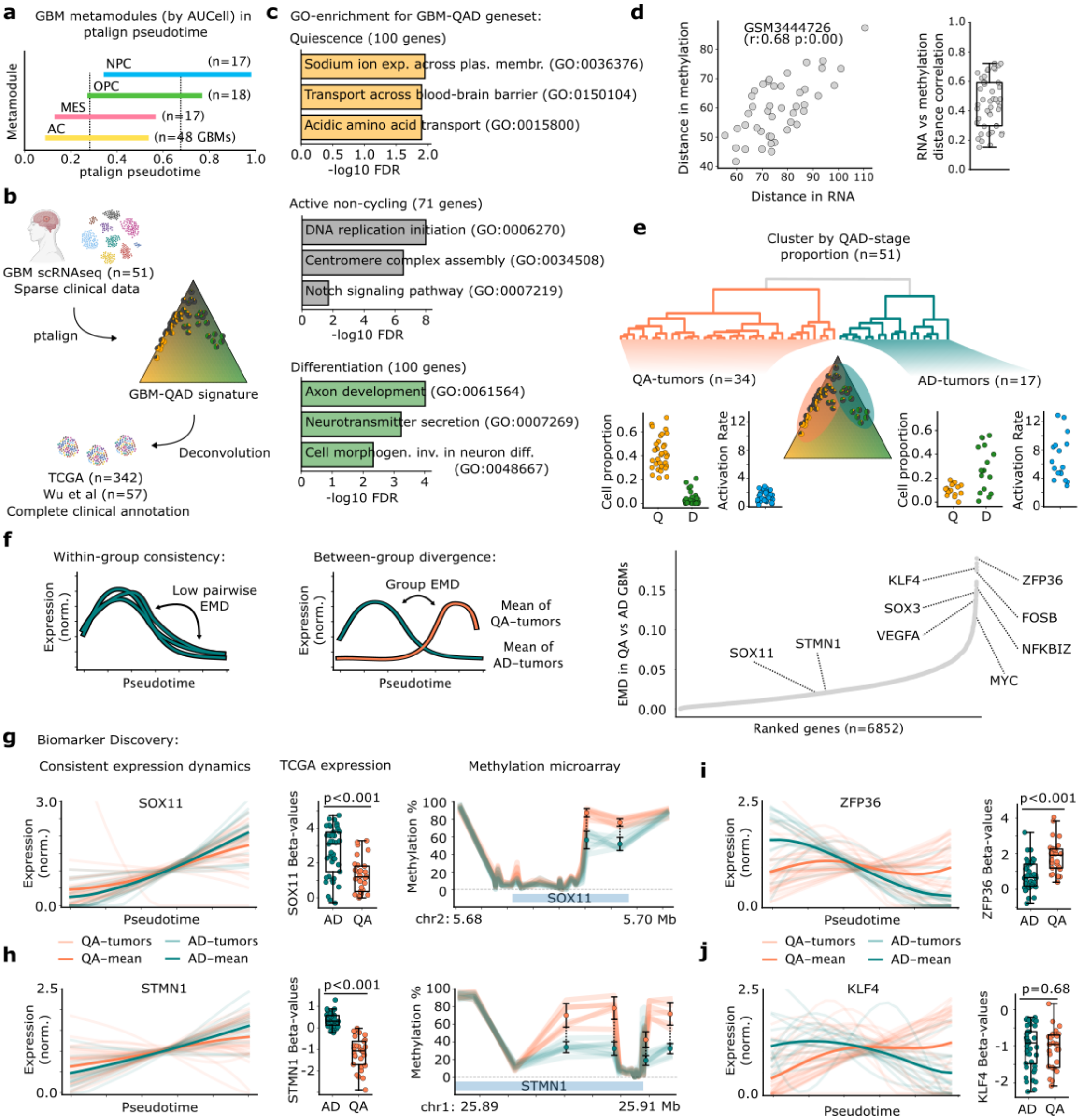

a
GBM metamodules (by AUCell) in ptalign pseudotime
Metamodule
NPC (n=17)
OPC (n=18)
MES (n=17)
AC (n=48 GBMs)
ptalign pseudotime
b
GBM scRNAseq (n=51)
Sparse clinical data
ptalign
GBM-QAD signature
Deconvolution
TCGA (n=342)
Wu et al (n=57)
Complete clinical annotation
c
GO-enrichment for GBM-QAD geneset:
Quiescence (100 genes)
Sodium ion exp. across plas. membr. (GO:0036376)
Transport across blood-brain barrier (GO:0150104)
Acidic amino acid transport (GO:0015800)
-log10 FDR
Active non-cycling (71 genes)
DNA replication initiation (GO:0006270)
Centromere complex assembly (GO:0034508)
Notch signaling pathway (GO:0007219)
-log10 FDR
Differentiation (100 genes)
Axon development (GO:0061564)
Neurotransmitter secretion (GO:0007269)
Cell morphogen. inv. in neuron diff. (GO:0048667)
-log10 FDR
d
GSM3444726
(r:0.68 p:0.00)
Distance in methylation
Distance in RNA
RNA vs methylation distance correlation
e
Cluster by QAD-stage proportion (n=51)
QA-tumors (n=34)
AD-tumors (n=17)
Cell proportion
Activation Rate
Q
D
f
Within-group consistency:
Low pairwise EMD
Expression (norm.)
Pseudotime
Between-group divergence:
Group EMD
Mean of QA-tumors
Mean of AD-tumors
EMD in QA vs AD GBMs
KLF4
SOX3
VEGFA
STMN1
SOX11
ZFP36
FOSB
NFKBIZ
MYC
Ranked genes (n=6852)
g
Biomarker Discovery:
Consistent expression dynamics
TCGA expression
Methylation microarray
SOX11
SOX11 Beta-values
p<0.001
AD
QA
Methylation %
chr2: 5.68
5.70 Mb
QA-tumors
AD-tumors
QA-mean
AD-mean
h
STMN1
STMN1 Beta-values
p<0.001
chr1: 25.89
25.91 Mb
i
ZFP36
ZFP36 Beta-values
p<0.001
j
KLF4
KLF4 Beta-values
p=0.68

Supplementary Fig. 6 | Leveraging expression heterogeneity in GBMs to identify genes with consistent and divergent expression dynamics

a Placement of GBM metamodules from Neftel et al[8] within ptalign pseudotime. Metamodule assignment by max AUCell > 0.2. Bars denote 90% highest-density interval, from averaging cell density across indicated number of GBMs with >10% of metamodule-assigned cells. **b** As scRNA-seq GBM datasets lack clinical metadata these are supplemented from bulk TCGA and Wu et al datasets through deconvolution. Created in BioRender. Kaya, O. (2025) https://BioRender.com/np19rtk. **c** Selected terms from GO-enrichment of GBM-QAD genes derived from n=51 scRNA-seq GBMs (see Methods). **d** Inter-tumoral euclidean distances in methylation- and RNA-modalities for a selected tumor from vs. other Wu et al[9] tumors (left) with Pearson correlation and p-value indicated; and across tumors (right). **e** Supporting patient stratification by clustering GBMs by QAD-stage, separating tumors into Q-biased (QA-) and D-biased (AD-) groups with varying activation rates in v-SVZ population models. **f** Left: Strategy for detecting group-specific expression dynamics: comparing between-group divergence while maximizing within-group consistency using the Earth Mover's Distance (EMD) for QA- and AD-groups from (e). Right: per-gene EMD average between QA- and AD-tumor groups. Relevant genes are indicated. **g-h** Supporting SOX11 (g) and STMN1 (h) as biomarkers for QA- vs AD-tumors. Each exhibits consistent pseudotime expression dynamics (left), group-specific expression in TCGA (center), and variable methylation (right). Methylation n=6 replicates in QA and n=8 in AD, from ellipses in Fig. 3g. Error bars in methylation panel denote standard deviation by group. P-values by two-sided t-test. **i-j** Expression splines for divergent QA- and AD-tumor genes ZFP36 (i, left) and KLF4 (j, left) and their group specific expression in TCGA (right). P-values by two-sided t-test. Box plots in c, f, h span 25th to 75th percentile, with median indicated. Whiskers extend to 1.5 times the interquartile-range. Source data are provided as a Source Data file.

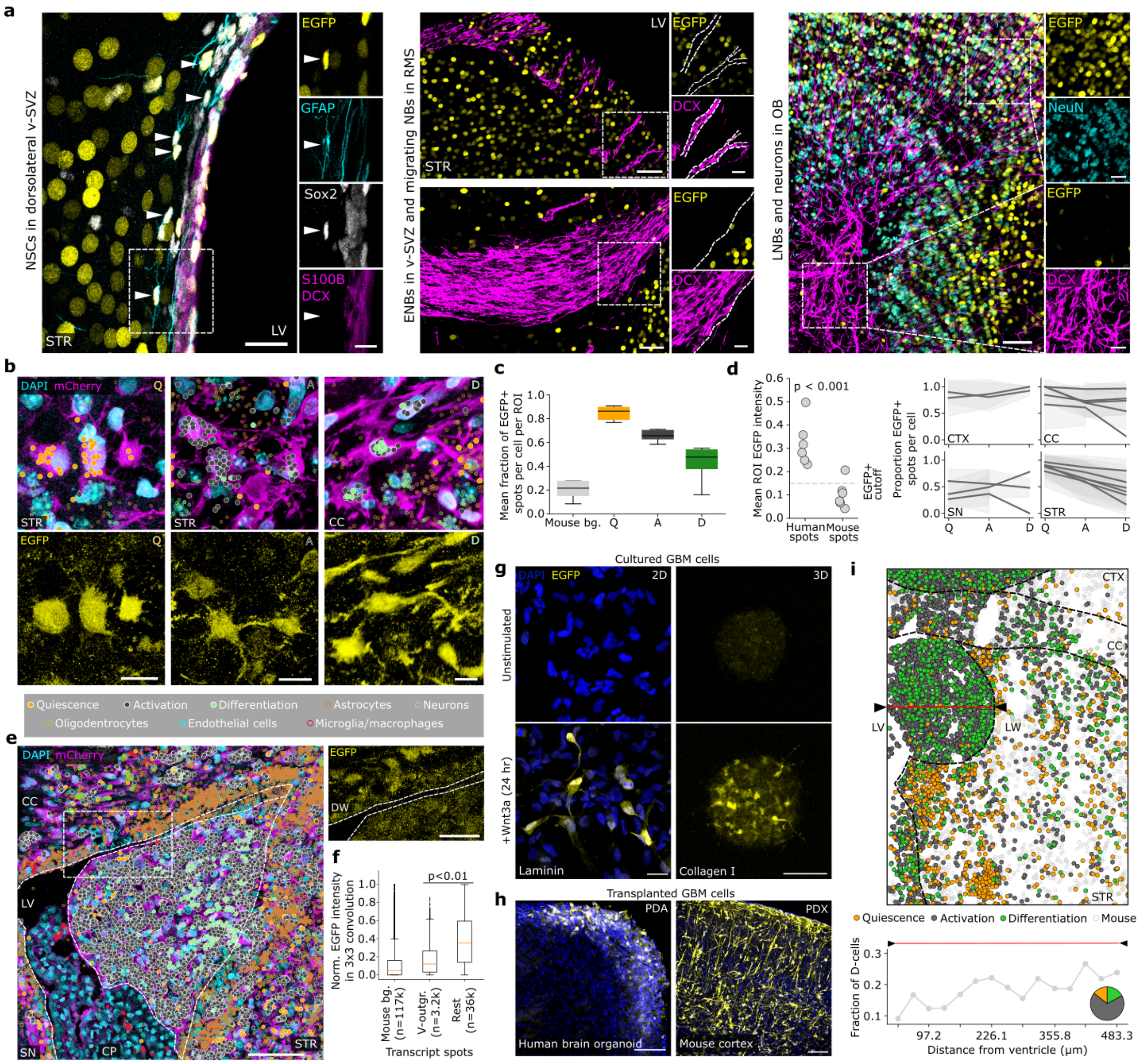

a
NSCs in dorsolateral v-SVZ
ENBs in v-SVZ and migrating NBs in RMS
LNBs and neurons in OB
EGFP
GFAP
Sox2
S100B
DCX
NeuN
STR
LV
b
DAPI mCherry
EGFP
Q
A
D
STR
CC
Quiescence
Activation
Differentiation
Astrocytes
Neurons
Oligodentrocytes
Endothelial cells
Microglia/macrophages
c
Mean fraction of EGFP+ spots per cell per ROI
Mouse bg.
d
p < 0.001
Mean ROI EGFP intensity
Human spots
Mouse spots
EGFP+ cutoff
Proportion EGFP+ spots per cell
CTX
SN
e
DW
CP
f
Norm. EGFP intensity in 3x3 convolution
p<0.01
Mouse bg. (n=117k)
V-outgr. (n=3.2k)
Rest (n=36k)
Transcript spots
g
Cultured GBM cells
DAPI EGFP
2D
3D
Unstimulated
+Wnt3a (24 hr)
Laminin
Collagen I
h
Transplanted GBM cells
PDA
PDX
Human brain organoid
Mouse cortex
i
LW
Mouse
Fraction of D-cells
Distance from ventricle (µm)
97.2
226.1
355.8
483.3

Supplementary Fig. 7 | Canonical Wnt signaling in healthy and malignant NSC lineages

a Full immunofluorescence images for Fig. 5, depicting TCF/Lef-H2B::EGFP activity for v-SVZ NSCs (left; coronal view; scale bars, 20 and 10µm), ENBs (NBs; center; sagittal view; scale bars, 50 and 20µm), and LNBs and neurons (right; sagittal view; scale bars, 50 and 20µm). Arrows identify v-SVZ NSCs by presence of GFAP, SOX2 and absence of S100B and DCX. **b** Selected Q (left), A (center), and D (right) cells in spatial transcriptomics with transcripts overlaid with DAPI and mCherry (top) or EGFP (bottom) immunofluorescence. Scale bars, 10µm. **c** Mean fraction of EGFP+ spots among pixels in mouse and QAD-stage cells for n=6 spatial transcriptomics ROIs. **d** EGFP+ spot threshold (left) and proportion of EGFP+ spots by QAD-stage in individual brain regions with >20 cells (right) from n=6 spatial transcriptomics ROIs. Significance was assessed by two-sided t-test, shaded area represents 90% confidence interval. **e** Left: Wnt-reporter spatial transcriptomics ROI of a ventricular outgrowth. Transcriptomic markers are overlaid with DAPI and mCherry immunofluorescence. Scale bar, 50µm. Right: EGFP fluorescence the ventricle edge. Scale bars, 25µm. **f** Normalized EGFP-intensity for mouse background (bg.) and tumor cells within (V-outgr.) and outside (rest) a ventricular growth seen in (e, n=1). Significance was assessed by permutation test. **g** Wnt-reporter GBM cells (EGFP+) on laminin coated chambers (left; scale bar, 25µm) or as 3D spheroids in collagen matrix (right; live, unstained; scale bar, 100µm), 24h post recombinant-Wnt3a treatment. **h** Wnt-reporter GBM cells (EGFP+) transplanted into HBOs (left, 10dpi) or mouse brains (right, 5mpi). Scale bars, 100µm. **i** Top: large ventricular growth in a non-Wnt-reporter spatial transcriptomics ROI with transcripts associated to segmented nuclei to assign species and QAD-stage. Red line traces largest extent from LW to LV. Bottom: increasing D-cell fraction in cells binned by distance to ventricle. Pie chart depicts QAD-stage proportions within the growth. ENB: early neuroblast; LNB: late neuroblast; STR: striatum; RMS: rostral migratory stream; OB: olfactory bulb; AC: astrocyte; CC: corpus callosum; CTX: cortex; SN: septal nuclei; LV: lateral ventricle; CP: choroid plexus; DW: dorsal-wall; d/mpi: days/months post injection. Box plots in c, f span 25th to 75th percentile, with median indicated. Whiskers extend to 1.5 times the interquartile-range. Source data are provided as a Source Data file.

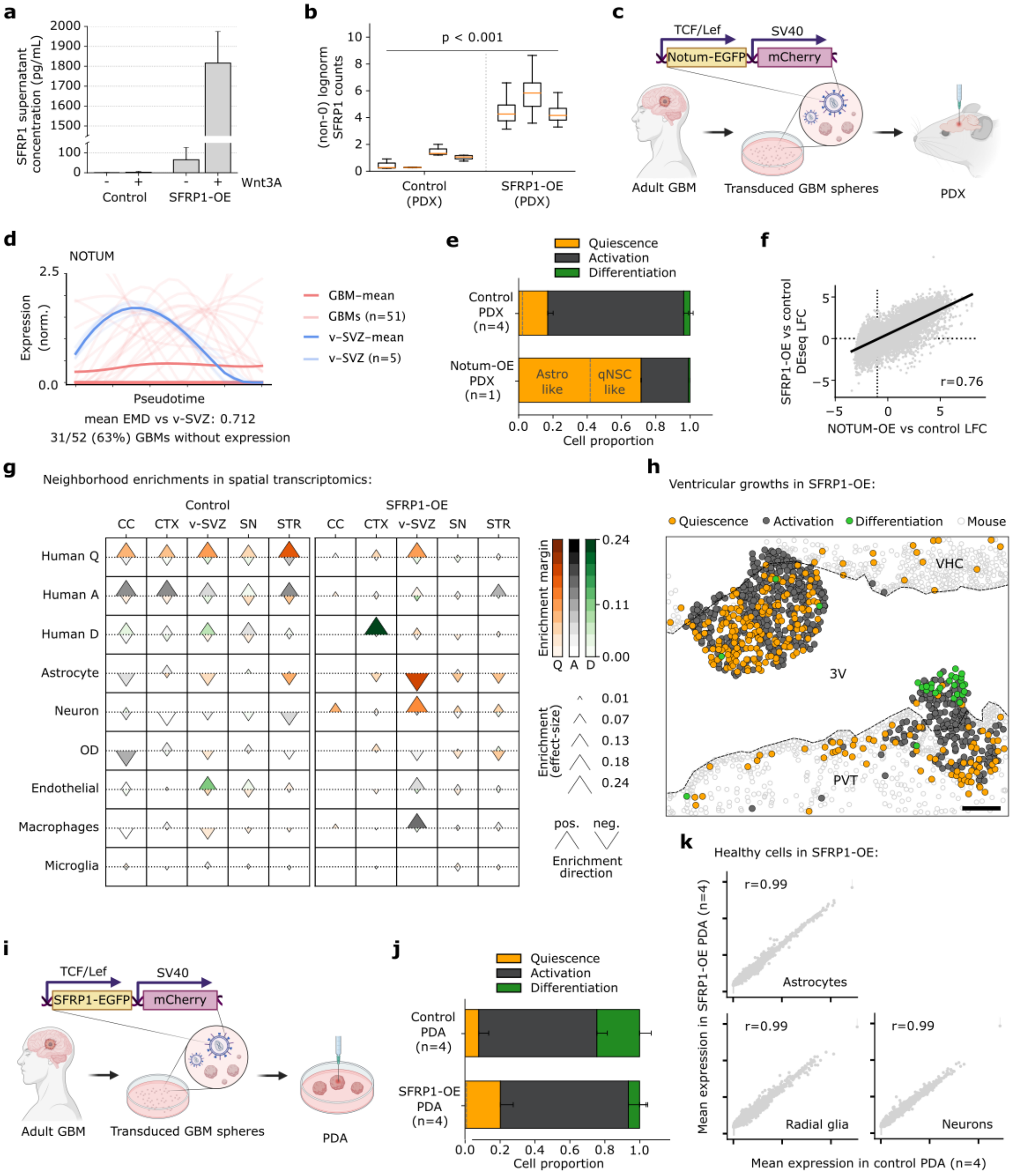
a
SFRP1 supernatant concentration (pg/mL)
Wnt3A
Control
SFRP1-OE
b
(non-0) lognorm SFRP1 counts
p < 0.001
Control (PDX)
SFRP1-OE (PDX)
c
TCF/Lef
SV40
Notum-EGFP
mCherry
Adult GBM
Transduced GBM spheres
PDX
d
NOTUM
Expression (norm.)
Pseudotime
GBM–mean
GBMs (n=51)
v-SVZ–mean
v-SVZ (n=5)
mean EMD vs v-SVZ: 0.712
31/52 (63%) GBMs without expression
e
Quiescence
Activation
Differentiation
Control PDX (n=4)
Notum-OE PDX (n=1)
Astro like
qNSC like
Cell proportion
f
SFRP1-OE vs control DEseq LFC
NOTUM-OE vs control LFC
r=0.76
g
Neighborhood enrichments in spatial transcriptomics:
Control
SFRP1-OE
CC
CTX
v-SVZ
SN
STR
Human Q
Human A
Human D
Astrocyte
Neuron
OD
Endothelial
Macrophages
Microglia
Enrichment margin
Q
A
D
Enrichment (effect-size)
pos.
neg.
Enrichment direction
h
Ventricular growths in SFRP1-OE:
Quiescence
Activation
Differentiation
Mouse
VHC
3V
PVT
i
TCF/Lef
SV40
SFRP1-EGFP
mCherry
Adult GBM
Transduced GBM spheres
PDA
j
Quiescence
Activation
Differentiation
Control PDA (n=4)
SFRP1-OE PDA (n=4)
Cell proportion
k
Healthy cells in SFRP1-OE:
Mean expression in SFRP1-OE PDA (n=4)
r=0.99
Astrocytes
Radial glia
Neurons
Mean expression in control PDA (n=4)

Supplementary Fig. 8 | Characterization of SFRP1-based intervention in preclinical models of GBM

a SFRP1 concentration by ELISA in supernatant of cells stimulated with/without Wnt3A. Bars present mean, error bars standard deviation in n=3 replicates. **b** Normalized SFRP1 expression levels in SFRP1-OE cells, by genotype. Significance was assessed by two-sided t-test. Box plots span 25th to 75th percentile, with median indicated. Whiskers extend to 1.5 times the interquartile-range. **c** NOTUM-OE construct used in T6 PDA tumor. mCherry ubiquitously labels tumor cells. **d** NOTUM dysregulation in n=51 tumors by expression splines as in Fig. 4. Excluded from previous analysis as most GBMs lose NOTUM expression. **e** Proportion of QAD-stage cells by ptalign in control (n=4 replicates) and NOTUM-OE (n=1) PDA cells. Bars present mean, error bars standard deviation. **f** NOTUM-OE log fold-change against SFRP1-OE DEseq2 log fold-change (LFC). Black line indicates best fit, Pearson correlation (r) is indicated. **g** Neighborhood enrichment by permutation (Methods) for segmented cells in n=17 Wnt-reporter and n=6 SFRP1-OE spatial transcriptomics ROIs. Cell type enrichments are reported for QAD-stage cells with other cell types (rows), with enrichment direction by shape and strength by size, while color relates the enrichment margin between the largest and second-largest enrichments. **h** Zoomed SFRP1-OE spatial transcriptomics ROI with QAD-stage cells in two ventricular outgrowths. Transcripts were associated to segmented nuclei to assign species and QAD-stage. Scale bar, 10µm. **i-j** SFRP1-OE construct used in T6 PDA tumors as in (c), with ptalign QAD-stage cells 14-dpi in SFRP1-OE (n=4 replicates) and control (n=4 replicates) PDA cells (j). Bars present mean, error bars standard deviation. **k** Mean log-normalized expression in control and SFRP1-OE PDA healthy astrocytes (left), radial glia (center) and neurons (right). Pearson correlation is indicated. OE: overexpressing; AC: astrocyte; RG: radial glia; CTX: cortex; CC: corpus callosum; SN: septal nuclei; STR: striatum; 3V: third ventricle; VHC: ventral hippocampal commissure; PVT: periventricular nuclei of the thalamus: dpi: days post injection. Panels c, i are created in BioRender. Kaya, O. (2025) https://BioRender.com/np19rtk. Source data are provided as a Source Data file.

References

1. Kalamakis, G. *et al.* Quiescence Modulates Stem Cell Maintenance and Regenerative Capacity in the Aging Brain. *Cell* **176**, 1407-1419.e14 (2019).
2. Kremer, L. P. M. *et al.* High throughput screening of novel AAV capsids identifies variants for transduction of adult NSCs within the subventricular zone. *Molecular Therapy - Methods & Clinical Development* **23**, 33–50 (2021).
3. Carvajal Ibañez, D. *et al.* Interferon regulates neural stem cell function at all ages by orchestrating mTOR and cell cycle. *EMBO Mol Med* **15**, e16434 (2023).
4. Nano, P. R. *et al.* A Meta-Atlas of the Developing Human Cortex Identifies Modules Driving Cell Subtype Specification. Preprint at https://doi.org/10.1101/2023.09.12.557406 (2023).
5. Jacob, F. *et al.* A Patient-Derived Glioblastoma Organoid Model and Biobank Recapitulates Inter- and Intra-tumoral Heterogeneity. *Cell* **180**, 188-204.e22 (2020).
6. Gavish, A. *et al.* Hallmarks of transcriptional intratumour heterogeneity across a thousand tumours. *Nature* **618**, 598–606 (2023).
7. Sumanaweera, D. *et al.* Gene-level alignment of single-cell trajectories. *Nat Methods* **22**, 68–81 (2025).
8. Neftel, C. *et al.* An Integrative Model of Cellular States, Plasticity, and Genetics for Glioblastoma. *Cell* **178**, 835-849.e21 (2019).
9. Wu, Y. *et al.* Glioblastoma epigenome profiling identifies SOX10 as a master regulator of molecular tumour subtype. *Nat Commun* **11**, 6434 (2020).
