# Supplementary material for: Cross-species comparison reveals therapeutic vulnerabilities halting glioblastoma progression
Source: Nat Commun. 2025 Aug 6;16:7250. doi: 10.1038/s41467-025-62528-w (PMC12329047; doi:10.1038/s41467-025-62528-w)
Supplement: Supplementary file 2 — Description of Additional Supplementary Files [file 41467_2025_62528_MOESM2_ESM.pdf]

Description of Additional Supplementary Files

File Name: Supplementary Data 1
Description: QAD ptalign gene sets and modeling parameters.

File Name: Supplementary Data 2
Description: Metadata for publicly available datasets.

File Name: Supplementary Data 3
Description: Population modeling details.

File Name: Supplementary Data 4
Description: QAD-predictive methylation probes and SFRP1-OE VMRs.

File Name: Supplementary Data 5
Description: Genes ranked by EMD.

File Name: Supplementary Data 6
Description: Spatial transcriptomics probes and spots.

File Name: Supplementary Data 7
Description: Antibodies and oligonucleotides.
