# Supplementary material for: Cross-species comparison reveals therapeutic vulnerabilities halting glioblastoma progression
Source: Nat Commun. 2025 Aug 6;16:7250. doi: 10.1038/s41467-025-62528-w (PMC12329047; doi:10.1038/s41467-025-62528-w)
Supplement: Supplementary file 10 — Reporting Summary [file 41467_2025_62528_MOESM10_ESM.pdf]

nature portfolio

Corresponding author(s): Ana Martin-Villalba

Last updated by author(s): 2025-07-22

Reporting Summary

Nature Portfolio wishes to improve the reproducibility of the work that we publish. This form provides structure for consistency and transparency in reporting. For further information on Nature Portfolio policies, see our Editorial Policies and the Editorial Policy Checklist.

Statistics

For all statistical analyses, confirm that the following items are present in the figure legend, table legend, main text, or Methods section.

n/a	Confirmed	
☐	☒	The exact sample size (*n*) for each experimental group/condition, given as a discrete number and unit of measurement
☐	☒	A statement on whether measurements were taken from distinct samples or whether the same sample was measured repeatedly
☐	☒	The statistical test(s) used AND whether they are one- or two-sided<br>*Only common tests should be described solely by name; describe more complex techniques in the Methods section.*
☐	☒	A description of all covariates tested
☐	☒	A description of any assumptions or corrections, such as tests of normality and adjustment for multiple comparisons
☐	☒	A full description of the statistical parameters including central tendency (e.g. means) or other basic estimates (e.g. regression coefficient) AND variation (e.g. standard deviation) or associated estimates of uncertainty (e.g. confidence intervals)
☐	☒	For null hypothesis testing, the test statistic (e.g. *F*, *t*, *r*) with confidence intervals, effect sizes, degrees of freedom and *P* value noted<br>*Give P values as exact values whenever suitable.*
☒	☐	For Bayesian analysis, information on the choice of priors and Markov chain Monte Carlo settings
☒	☐	For hierarchical and complex designs, identification of the appropriate level for tests and full reporting of outcomes
☐	☒	Estimates of effect sizes (e.g. Cohen's *d*, Pearson's *r*), indicating how they were calculated

Our web collection on statistics for biologists contains articles on many of the points above.

Software and code

Policy information about availability of computer code

Data collection	Data collection did not depend on software.
Data analysis	Raw sequencing data were processed using STAR v2.5.3a, trim-galore v0.5.0, samtools v1.5, cellranger v6.0.0, and CITE-seq count v1.4.5, with BLAST+ v2.2.30 and mygene v1.8 using in TCGA data curation. WGBS mappings were carried out with Bismark v0.20 and Bowtie v2.3.5.1. Baysor v0.5.2, squidpy v1.5.0, cellpose v.3.0.10 and scikit-image were used in spatial transcriptomics and/or image analysis. Image analysis was done using Image J Version v2.1.0/1.53c and Leica Application Suite X v1.4.4. Data analysis was carried out in jupyter notebooks running python 3.7 including the standard numpy 1.23.3, pandas 1.1.4, scipy 1.9.1, and scikit-learn 1.1.1 packages. Single-cell analysis was done in scanpy 1.9.1. Also used were statsmodels 0.12.2, kneed 0.7.0, lifelines 0.26.4, and gseapy 0.10.8. Spatial transcriptomics used tifffile 2022.5.4, readlif 0.6.5, Pillow 8.2.0, opencv-python 4.7.0.68, scikit-image 0.19.2, DeepCell 0.12.2, and squidpy 1.2.3. Plotting used matplotlib 3.4.2, python-ternary 1.0.8, seaborn 0.11.1, and brokenaxes 0.5.0. Remaining analyses were conducted in R v3.6.1, using DEseq2 1.24.0, inferCNV 1.2.1, Seurat 3.1.0, msigdbr 7.0.1, and GSVA 1.34.0. Custom code developed for this study include an AUCell implementation based on pyscenic, and a SmartSeq3 UMI-counting tool based in HTseq. Custom code from this study is available on zenodo: https://doi.org/10.5281/zenodo.8186500 for spatial transcriptomics and https://doi.org/10.5281/zenodo.14968415 the rest. Detailed description of our analysis is provided in the Methods. The ptalign software is available on GitHub https://github.com/leoforster/ptalign.

For manuscripts utilizing custom algorithms or software that are central to the research but not yet described in published literature, software must be made available to editors and reviewers. We strongly encourage code deposition in a community repository (e.g. GitHub). See the Nature Portfolio guidelines for submitting code & software for further information.

Data

Policy information about availability of data

All manuscripts must include a data availability statement. This statement should provide the following information, where applicable:

- Accession codes, unique identifiers, or web links for publicly available datasets
- A description of any restrictions on data availability
- For clinical datasets or third party data, please ensure that the statement adheres to our policy

Newly generated SmartSeq3 datasets of the mouse v-SVZ NSCs lineage are available from GEO with accession GSE240676 [https://www.ncbi.nlm.nih.gov/geo/query/acc.cgi?acc=GSE240676], along with processed tumor PDX and PDA single-cell RNA-sequencing and WGBS datasets. Raw tumor data subject to DPA are submitted to EGA with accession EGAS00001008155 [https://ega-archive.org/studies/EGAS00001008155]. Access to the data is controlled due to ethical and privacy considerations related to human-derived samples, in compliance with data protection regulations. Access will be granted to qualified researchers for non-commercial academic use following approval by the Data Access Committee. Researchers should submit a data access request via the EGA portal, which will be evaluated based on the proposed use. Requests will be reviewed and data made available as outlined in the DFKZ Data Transfer Agreement [https://metadata.ega-archive.org/policies/EGAP00001002156]. Generated spatial transcriptomics data are submitted to Zenodo under accession code 8186500 [https://doi.org/10.5281/zenodo.8186500]. Analyzed GBM scRNA-seq datasets which had been previously published were retrieved as follows: Richards et al8 (SCP503 [https://singlecell.broadinstitute.org/single_cell/study/SCP503]), Bhaduri et al5 (personal communication), Neftel et al4 (SCP393 [https://singlecell.broadinstitute.org/single_cell/study/SCP393]), Yuan et al48 (GSE103224 [https://www.ncbi.nlm.nih.gov/geo/query/acc.cgi?acc=GSE103224]), Couturier et al6 (GitHub [https://datahub-262-c54.p.genap.ca/GBM_paper_data/GBM_cellranger_matrix.tar.gz]), Wang L. et al114 (GSE138794 [https://www.ncbi.nlm.nih.gov/geo/query/acc.cgi?acc=GSE138794]), Jacob et al39 (GSE141946 [https://www.ncbi.nlm.nih.gov/geo/query/acc.cgi?acc=GSE141946]), Wang R. et al50 (GSE139448 [https://www.ncbi.nlm.nih.gov/geo/query/acc.cgi?acc=GSE139448]), and Chen et al51 (GSE141383 [https://www.ncbi.nlm.nih.gov/geo/query/acc.cgi?acc=GSE141383]). Previously published scRNA-seq data of the v-SVZ which we re-analyzed in this study were retrieved from the following sources: Cebrian-Silla et al90 (https://cells.ucsc.edu/?ds=svzneurogeniclineage), Kremer et al34 (GSE145172 [https://www.ncbi.nlm.nih.gov/geo/query/acc.cgi?acc=GSE145172]), Carvajal-Ibanez et al35 (GSE197217 [https://www.ncbi.nlm.nih.gov/geo/query/acc.cgi?acc=GSE197217]), and Kalamakis et al15 (GSE115626 [https://www.ncbi.nlm.nih.gov/geo/query/acc.cgi?acc=GSE115626]). Samples and metadata from the TGCA-GBM cohort are available through the NCI GDC Data Portal (https://portal.gdc.cancer.gov/). Bulk GBM data from Wu et al20 was received in personal communication. Source data are provided with this paper. All data supporting the findings of this study are available from the corresponding author upon reasonable request.

Research involving human participants, their data, or biological material

Policy information about studies with human participants or human data. See also policy information about sex, gender (identity/presentation), and sexual orientation and race, ethnicity and racism.

Reporting on sex and gender	Supplementary Data 2 presents pseudonymized clinical data for the patient cohort we curated based on previously published studies. The data include non-identifiable clinical information, as well as patient age and sex. No additional identifiers (such as exact birth dates, rare diagnoses, or medical center information) are disclosed. Therefore, specific consent for publication of identifying information was not required. Additionally, primary tumor material from one patient was obtained under informed consent prior to surgery at the University Hospital Ulm. All experiments involving human tissue were approved by the ethics committees of Ethics Commission of the Medical Faculty Heidelberg (S-224/2021) and conducted in accordance with the Declaration of Helsinki. No identifiable information from this patient is disclosed in the manuscript.
Reporting on race, ethnicity, or other socially relevant groupings	See above.
Population characteristics	Described in Supplementary Data 2
Recruitment	Based on availability.
Ethics oversight	Primary tumor samples were received from the University Hospital Ulm upon obtaining informed consent prior to surgery. Experiments involving patient tumor biopsies were carried out in accordance with the Declaration of Helsinki and were approved by the ethics committees of Ethics Commission of the Medical Faculty Heidelberg (S-224/2021). All tumor specimens were examined by a neuropathologist to ensure that the tumors met GBM criteria defined by the World Health Organization.

Note that full information on the approval of the study protocol must also be provided in the manuscript.

Field-specific reporting

Please select the one below that is the best fit for your research. If you are not sure, read the appropriate sections before making your selection.

☒ Life sciences ☐ Behavioural & social sciences ☐ Ecological, evolutionary & environmental sciences

For a reference copy of the document with all sections, see nature.com/documents/nr-reporting-summary-flat.pdf

Life sciences study design

All studies must disclose on these points even when the disclosure is negative.

Sample size	No statistical methods were used to predetermine sample size. Conventional cohort sizes were used in PDX models to account for expected animal-to-animal variability; for PDA models replication was limited by the number of available organoids.
Data exclusions	No data were excluded in the course of the experiments carried out in the proposed study design. Data analysis effected the exclusion of low quality cells and datasets, along with low-quality TCGA samples or those having undergone treatment (i.e. recurrent tumors) or with a prior malignancy.
Replication	All experiments were carried out in three or more replicates. Biological and technical replicates validating obtained experimental results were employed and specified at respective sections.
Randomization	No randomization was used in the course of this study.
Blinding	Investigators were not blinded to the experimental conditions.

Reporting for specific materials, systems and methods

We require information from authors about some types of materials, experimental systems and methods used in many studies. Here, indicate whether each material, system or method listed is relevant to your study. If you are not sure if a list item applies to your research, read the appropriate section before selecting a response.

Materials & experimental systems

n/a	Involved in the study	
☐	☒	Antibodies
☐	☒	Eukaryotic cell lines
☒	☐	Palaeontology and archaeology
☐	☒	Animals and other organisms
☒	☐	Clinical data
☒	☐	Dual use research of concern
☒	☐	Plants

Methods

n/a	Involved in the study	
☒	☐	ChIP-seq
☐	☒	Flow cytometry
☒	☐	MRI-based neuroimaging

Antibodies

Antibodies used	Data on Antibody supplier, catalog, clone, and lot are given in Supplementary Data 7.
Validation	Only antibodies validated by the manufacturer or those used in previously published studies were used in this study.

Eukaryotic cell lines

Policy information about cell lines and Sex and Gender in Research

Cell line source(s)	Human iPSCs were acquired from Allen Institute for Cell Science (AICS-0036-006, https://www.allencell.org/cell-catalog.html). The AICS-0036-006 line was labeled with EGFP via CRISPR/Cas9 system, where mEGFP transgene was knocked in chromosome 19q13-qter. Their parental cell line (https://www.coriell.org, GM25256) was acquired from Coriell (UCSFi001-A, https://labs.gladstone.org/conklin/) and was derived from a healthy donor using an episomal reprogramming method. For patient tumor samples, fresh tissues were immediately dissociated using Brain Tumor Dissociation Kit (P) and expanded in culture upon arrival. The single line used in this study (T6) originated from a male patient. See Methods.
Authentication	The cell lines used were not authenticated.
Mycoplasma contamination	The cell lines used were not tested for mycoplasma contamination.
Commonly misidentified lines (See ICLAC register)	We used human embryonic kidney 293 cells for the generation and titration of lentiviral particles, which does not involve direct experimentation on the cell line per se. Otherwise, no other commonly misidentified cell line was used in the study.

Animals and other research organisms

Policy information about studies involving animals; ARRIVE guidelines recommended for reporting animal research, and Sex and Gender in Research

Laboratory animals	Male TCF/Lef:H2B/GFP mice2 were bred at the Center for Preclinical Research at the German Cancer Research Center and were used

	for analyzing TCF/Lef-activity in mouse NSC studies. Male Fox Chase SCID Beige mice (CB17.Cg-PrkdcscidLystbg-J/Crl) were purchased from Charles River and were used to generate human-mouse xenograft tumors. See Methods.
Wild animals	The study did not involve wild animals.
Reporting on sex	To be consistent with the previously published datasets from our research group, only male mice were used for sequencing experiments.
Field-collected samples	The study did not involve field-collected samples.
Ethics oversight	All procedures were approved and conform to the regulatory guidelines of the official committee (Regierungspräsidium Karlsruhe, Germany; G19/21).

Note that full information on the approval of the study protocol must also be provided in the manuscript.

Flow Cytometry

Plots

Confirm that:

☐ The axis labels state the marker and fluorochrome used (e.g. CD4-FITC).

☐ The axis scales are clearly visible. Include numbers along axes only for bottom left plot of group (a 'group' is an analysis of identical markers).

☐ All plots are contour plots with outliers or pseudocolor plots.

☐ A numerical value for number of cells or percentage (with statistics) is provided.

Methodology

Sample preparation	Described in Methods.
Instrument	BD Fortessa, BD FACSAria II and BD FACSAria Fusion.
Software	BD FACSDiva v9.0
Cell population abundance	Populations of interest were validated through single cell RNA sequencing.
Gating strategy	The gating on healthy adult neural stem cells was performed as previously reported (Kalamakis et al., Cell, 2019).

☐ Tick this box to confirm that a figure exemplifying the gating strategy is provided in the Supplementary Information.
